# Phosphate Frustration: Treatment Options to Complement Current Therapies

**DOI:** 10.1155/2022/9457440

**Published:** 2022-08-22

**Authors:** Pablo E. Pergola

**Affiliations:** Renal Associates, PA, San Antonio, TX 78212, USA

## Abstract

Hyperphosphatemia eventually develops in almost all patients with advanced chronic kidney disease and is associated with negative clinical outcomes. Thus, guidelines recommend targeting treatment to normal phosphate levels in patients with chronic kidney disease. Despite low phosphorus diets, clearance by dialysis, and phosphate binder use, many patients with chronic kidney disease on dialysis are unable to consistently achieve and maintain serum phosphate concentrations <5.5 mg/dL. A chart audit of patients on dialysis receiving phosphate binders showed that 74 to 86% were unable to consistently achieve serum phosphate ≤5.5 mg/dL over 6 months. Furthermore, although there is evidence that serum phosphate concentrations <4.5 mg/dL are associated with improved survival and cardiovascular outcomes, real-world phosphate control data suggest achieving and maintaining this goal for most patients would be extremely challenging, if not near impossible, using current therapies. As phosphate binders can only remove approximately 300 mg of the 2,500 mg or more daily dietary phosphate intake, therapeutic innovations are necessary to improve phosphate management. We present treatment options to complement current therapies including tenapanor, a novel sodium/hydrogen exchanger isoform 3 inhibitor that blocks the dominant paracellular phosphate absorption pathway and has been shown to reduce phosphate levels in several clinical trials.

## 1. Importance of Phosphate Regulation in Chronic Kidney Disease

Phosphorus is one of the most common minerals in the body. Normal phosphate concentrations are essential for the proper function of many biological processes, including cellular energy production, the release of oxygen to peripheral tissues by red blood cells, and bone mineralization [1]. Chronic kidney disease (CKD) affects approximately 37 million people in the US, and elevated phosphate levels are often seen in later CKD stages due to impaired kidney function [2, 3]. Parathyroid hormone (PTH) and fibroblast growth factor-23 (FGF23) levels rise in the early stages of CKD and increase phosphate excretion to maintain normal serum phosphate concentrations [4–6]. However, as kidney function worsens, these compensatory mechanisms eventually become insufficient to keep serum phosphate levels from rising [4]. By recent estimates, 83% of patients with CKD on dialysis are treated for hyperphosphatemia. [3].

Chronic hyperphosphatemia is associated with numerous disease processes like vascular calcification, cardiovascular (CV) disease (CVD), and secondary hyperparathyroidism [7–9]. In patients with CKD, abnormal phosphate levels are an independent risk factor for CV morbidity and mortality [10–12]. This is particularly important as CVD accounted for over 50% of deaths among patients with CKD on dialysis in 2018 [13]. Higher serum phosphate concentrations were associated with both all-cause and CV mortality in adjusted analyses. A study of patients on dialysis found that serum phosphate concentrations >5.0 mg/dL were associated with an increased relative risk of death up to 102% [14]. There is also evidence suggesting that poor phosphate control, or serum phosphate concentrations >4.5 mg/dL over a 6-month period, is associated with CV mortality, while phosphate concentrations <4.5 mg/dL are correlated with improved survival [15]. In patients with CKD not on dialysis, every 1 mg/dL increase in serum phosphate was shown to be associated with an increased risk of both kidney failure (HR: 1.36; 95% CI: 1.20 to 1.55; *p* < 0.01) and mortality (HR: 1.20; 95% CI: 1.05 to 1.37; *p*=0.02) [16]. Phosphate retention has even been shown to negatively impact health in adults without CKD; a study of 12,984 participants without CKD showed that every 1 mg/dL increase over 3.5 mg/dL was associated with 35% increased risk of all-cause death (HR: 1.35; 95% CI: 1.05 to 1.74; *p*=0.02) and a 45% increased risk of CV death (HR: 1.45; 95% CI: 1.05 to 2.00; *p*=0.03) after adjusting for examination session (morning vs. afternoon/evening), age, gender, African-American race, Mexican ethnicity, poverty, inactivity, BMI (knot at 20 kg/m^2^), smoking status, systolic blood pressure, diabetes, non-high density lipoprotein cholesterol (knot at 100 mg/dL), ln (ACR), eGFR, and vitamin D status [17].

Given the negative clinical outcomes in patients with elevated serum phosphate concentrations, lowering them towards normal levels is a standard clinical practice. This is supported by guideline recommendations [18, 19] For example, KDOQI/NKF guidelines recommend that patients with stage 3 and 4 CKD aim for phosphate levels from 2.7 to 4.6 mg/dL, and patients with stage 5 CKD target 3.5 to 5.5 mg/dL. The 5.5 mg/dL target level was included in the KDOQI guideline based on opinions from experts; however, they recognized that the target is ideally lower [18]. Furthermore, the KDIGO guidelines published in 2017 recommend lowering elevated phosphate levels towards the normal range based on a systematic review of relevant trials [19]. The KDIGO guidelines do note a trial that observed the best patient survival with serum phosphate levels close to 4.4 mg/dL [19, 20].

## 2. Suboptimal Phosphate Control in Current Practice

**Figure 1:**
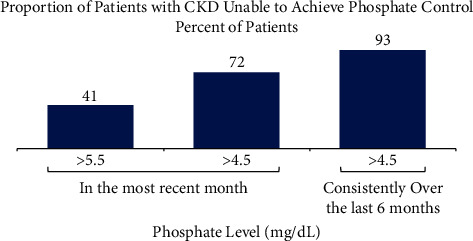
Percent of patients with CKD unable to achieve phosphate control [15, 22]. Many patients on dialysis are unable to consistently achieve and maintain target phosphate concentrations, let alone more normal levels of <4.5 mg/dL. Almost all patients on dialysis cannot consistently achieve and maintain phosphate concentrations <4.5 mg/dL over 6 months.

Although approximately 80% of patients on dialysis in the US are prescribed phosphate binders to manage hyperphosphatemia [3], and dietary counseling is standard practice for patients on dialysis [21], a significant proportion of these patients are unable to consistently achieve and maintain serum phosphate levels ≤5.5 mg/dL, let alone more normal levels of ≤4.5 mg/dL. Based on data from the Dialysis Outcomes and Practice Patterns Study (DOPPS), 41% and 72% of patients had serum phosphate levels >5.5 mg/dL and >4.5 mg/dL in the most recent month, respectively (Figure 1) [22], and 93% were unable to consistently achieve serum phosphate levels <4.5 mg/dL over 6 months [15].

Phosphate binders, drugs that work in the gastrointestinal (GI) tract to create nonabsorbable compounds that are excreted in the stool, are the only currently available pharmacological therapy for hyperphosphatemia [23–28]. Aluminum binders, introduced in the 1970s, were the first of this class [29]. Although they were effective, their use was largely discontinued in the 1980s due to an association with neurotoxicity [30], cognitive disturbances, osteomalacia, and anemia [31]. Calcium-based binders widely used as a replacement for aluminum binders were found to be potential drivers of vascular calcification and [32], thus, contributors to increased CV mortality [33]. Sevelamer hydrochloride and lanthanum carbonate were approved in 2000 and 2004, respectively [25, 27]. These binders do not increase risk of calcium overload and are frequently used to manage hyperphosphatemia [25]. The iron-based binders sucroferric hydroxide and ferric citrate were approved in 2013 and 2014, respectively [24, 26]. No other drug classes have been approved for phosphate management since then with no advances within the phosphate binder class in nearly a decade.

Clinical trial data show that phosphate binders are effective in reducing phosphate levels (Table 1). Despite the efficacy illustrated by these studies, even in the selected clinical trial populations of patients on dialysis, followed under strict protocol requirements and provided access to free drug, phosphate binder use failed to achieve and maintain more normal phosphate levels of ≤4.5 mg/dL and in many cases not even ≤5.5 mg/dL [27, 34, 35, 39, 40].

**Figure 2:**
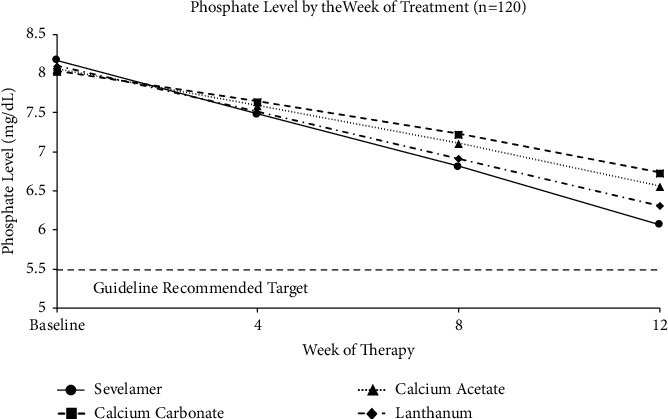
Current therapies fail to lower phosphate to target levels [42]. A comparative study of calcium acetate, calcium carbonate, sevelamer hydrochloride, and lanthanum carbonate was conducted in 120 patients with ESRD on dialysis [42]. A total of 30 patients were randomly assigned to each binder type [42]. Reductions in serum phosphorus were observed for all phosphate binders but on average did not get below 5.5 mg/dL.

Beyond clinical trials, real-world chart audit data showed that the majority of binder-treated patients on dialysis were unable to consistently achieve serum phosphorus concentrations ≤5.5 mg/dL over 6 months [41]. The inability to achieve and maintain consistent phosphate control shown was similar regardless of the binder type; 77% of patients prescribed various types of phosphate binders had a maximum serum phosphate value >5.5 mg/dL in the past 6 months [41]. Similarly, a study comparing phosphate binders in patients on dialysis showed that although they were effective in lowering serum phosphate levels, phosphate binder therapy alone was insufficient to achieve target phosphate levels of <5.5 mg/dL over the 12-week study period (Figure 2) [42].

Current phosphate management strategies are generally insufficient to match daily dietary phosphorus intake [43]. Binding capacities for the recommended starting daily dose of each binder range from 126 to 403 mg of phosphorus. Binding capacity of different compounds is as follows:

1. Lanthanum carbonate: 135 mg phosphorus per 1,000 mg tablet × 1500 mg recommended daily dose = 203 mg phosphorus per day and × 3000 mg for the maximal recommended daily dose = 403 mg phosphorus per day [25, 44, 45]
2. Sevelamer: 21 mg phosphorus per 800 mg capsule × 4800 mg recommended daily dose = 126 mg phosphorus per day and × 14,400 mg for the maximal recommended daily dose = 378 mg phosphorus per day [27, 44]
3. Calcium acetate: 44 mg phosphorus per 1000 mg of medication × 4002 mg recommended daily dose = 176 mg phosphorus per day and × 8,004 mg for the maximal recommended daily dose = 352 mg phosphorus per day [23, 45, 46]

**Figure 3:**
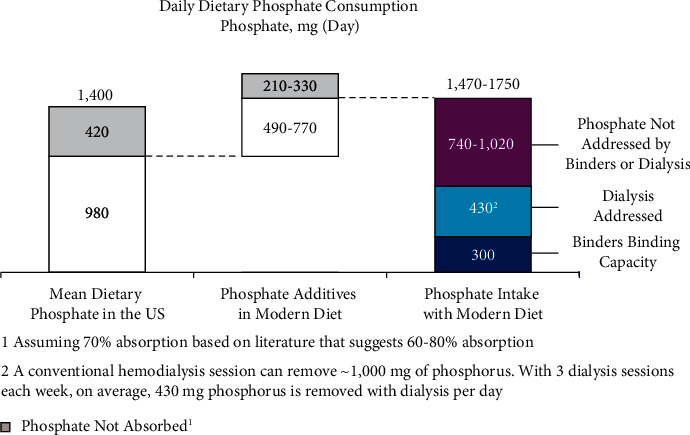
Only a small proportion of dietary phosphate is addressed by binders and dialysis [23, 25, 27, 44–46, 52–55]. Phosphate binders and dialysis can address ∼730 mg of phosphate per day (for example, an average of 300 mg/day of phosphorus binding was used for binders and not the potential maximum of 400 mg/day). However, this is far less than the mean daily dietary phosphate consumption in the US (∼1400 mg), particularly for diets high in phosphate additives, which can add ∼1,100 mg phosphate. Therefore, over 1,000 mg phosphate per day may not be addressed by binders and dialysis, increasing the risk of hyperphosphatemia and associated negative clinical outcomes. A net neutral phosphate balance can be achieved if patients strictly follow the guideline-recommended restricted phosphate intake and avoid most phosphate additives.

Doses for each binder may be titrated up to achieve better phosphate control [23–27], but side effects associated with binders may be dose limiting. Gastrointestinal upset is a common side effect of many types of binders [47]. Guidelines recommend that calcium-based binders be dose-restricted or avoided due to the risk of hypercalcemia and the concern that calcium-based binders, even in the presence of normal serum calcium levels, may accelerate vascular calcification [48–50]. Even so, high doses of phosphate binders requiring multiple tablets/capsules per meal can typically only remove up to 400 mg of phosphorus per day, as illustrated above [51]. As the mean daily dietary phosphorus load in the US may be as high as 2,500 mg (∼1,400 mg based on dietary databases with an additional ∼1,100 mg from phosphate additives) [51], over 1,000 mg of dietary phosphate may not be addressed by phosphate management strategies; the maximum 400 mg binding capacity in conjunction with 430 mg phosphorus removed by dialysis is only sufficient to match a daily dietary phosphate load of 830 mg (Figure 3) [52]. Binding capability may be further limited by systemic pH [56]. A study assessing the quantity of bound phosphate for 5 binders in solutions at 2 pH values found that 4 binders (lanthanum carbonate, sevelamer carbonate, calcium carbonate, and sucroferric oxyhydroxide) were more effective in a solution with a baseline pH of 3, and 1 (calcium acetate/magnesium carbonate) was the most effective with a baseline pH of 6 [56]. Although these data are taken from in vitro experiments [56], they suggest that phosphate binder efficacy may vary based on individual patients' intestinal pH. Additional potential mechanistic explanations for suboptimal phosphate control may be that binders have low in vivo binding capacities due to nonspecific binding [44, 45] and short durations of action that require patients to take binders with each meal or snack, leading to imperfect dosing adherence. Furthermore, binders do not directly target or act on either of the two main phosphate absorption pathways [23–27]. First, it is understood that intestinal phosphate absorption takes place via two distinct pathways [57–60]. In the paracellular pathway, phosphate is absorbed along concentration gradients through tight junction protein complexes (e.g., claudins and occludin), consistent with passive diffusion [61–63]. In the transcellular pathway, active phosphate uptake occurs predominantly through the sodium-dependent phosphate cotransporter 2b (NaPi2b) [64]. Recent studies have shown that the paracellular pathway is the dominant mechanism for phosphate absorption in humans [63, 65, 66]. The phosphate binder mechanism of action only indirectly impacts these pathways. Because binders must simultaneously be present in the GI tract with dietary phosphorus to work, they are unable to impact phosphorus absorption when a dose is missed or not properly timed [23–27], significantly increasing the risk of serum phosphate concentrations >5.5 mg/dL [67, 68]. Moreover, because each pill can only bind a certain amount of phosphorus, patients are typically required to ingest large and/or many pills per dose, with increasing pill requirements as dietary phosphorus intake increases [23–27]. Thus, many physicians feel it is extremely challenging to help patients consistently maintain levels <5.5 mg/dL and even more difficult for them to achieve more normal levels of <4.5 mg/dL.

## 3. Impact of Phosphorus Management Strategies and Suboptimal Phosphate Control on Patients' Quality of Life and Providers' Attitude

The phosphate binder dosing requirement (number and size of pills and dosing frequency), adverse effects associated with binders, and the clinical pressure to achieve target serum phosphate levels can negatively impact patient experience and the patient/clinician relationship. Patients have reported the difficulty, dissatisfaction, and inconvenience of taking phosphate binders [69]. Specific contributors to dissatisfaction or lower quality of life include high pill burden [70], large tablet size [71], bad taste [71], and negative side effects [71, 72]. Many patients do not fully understand the importance of phosphate control, and this lack of understanding likely increases their frustration with the negative impact of phosphate binders on the quality of life [73]. A study of dialysis providers' attitudes towards phosphate binder therapy found that practitioners did not endorse phosphate binders as the most important medication for dialysis patients, nor did they believe that phosphate binders are more important than diet for phosphate control [74]. Dialysis providers also had limited confidence in patients' ability to make any changes regarding phosphate binder use [74]. However, providers did feel highly responsible for addressing nonadherence to phosphate binders [74]. The combination of pointing out nonadherence and lack of confidence in patients' ability to self-manage may negatively impact the patient/clinician relationship because patients may feel like they are being criticized and that providers are condescending. It is important to note that these frustrations on both patients and providers stem primarily from the inability of phosphate binders, dialysis, and diet to help consistently achieve and maintain target phosphate levels because of the inherent limitations of the therapy (as discussed above) but not from any fault of patients or providers.

Both the cost of binders and their inability to help consistently achieve and maintain target phosphate levels also have a significant economic impact. In 2015, Medicare costs for phosphate binders for patients with CKD on dialysis in the United States exceeded $1.5 billion, and costs for CKD-mineral bone disease medications have increased faster than other medication categories in this patient population [75]. An evaluation of the improved “percent time in the range” for target serum phosphate concentrations found that increasing the percent time in the range by 60% results in a 6% reduction in inpatient costs. [76] As increased phosphate concentrations drive increases in PTH levels and subsequent disturbances of calcium and vitamin D, it is logical that improved phosphate control would translate to improvements in secondary hyperparathyroidism and CKD-mineral bone disease. Annual direct medication costs for treating secondary hyperparathyroidism (∼$10 000 to $16 000 per patient) would likely be reduced given proper phosphate control [77]. Thus, improved phosphate control could lead to significant cost savings even if not taking into account potential long-term improvements in CV outcomes.

## 4. Novel Therapies to Achieve Improved Phosphate Control

The continuing clinical challenge of phosphate control points to a need for therapeutic innovation. Tenapanor is an investigational nonbinder phosphate absorption inhibitor that blocks paracellular phosphate absorption in the GI tract and thus targets the primary absorption pathway, providing a novel approach to treating hyperphosphatemia [65]. Local inhibition of the sodium/hydrogen exchanger isoform 3 (NHE3) by tenapanor directly reduces sodium absorption, leading to modest intracellular proton retention that is proposed to induce conformational changes in tight junction proteins [65]. These changes directly reduce permeability specific to phosphate through the paracellular pathway [65]. By blocking the primary pathway of phosphate absorption, tenapanor acts directly to reduce serum phosphate concentrations [65, 78–80]. Tenapanor's unique mechanism of action allows the drug to be active for several hours after administration at low doses (e.g., 10 to 30 mg) [80, 81], compared with several thousand mg per day required for phosphate binders [23–27]. A study also found that adding tenapanor (30 mg twice daily orally) to phosphate binders reduced the binder pill burden from 15 to 3 tablets per day, and 72% of patients achieved a ≥30% decrease in the total pill burden by the end of the 26-week treatment period (*p* < 0.001) [82]. 29% of patients were switched completely to tenapanor (*p* < 0.001), and phosphorus levels were effectively controlled in all patients [82].

Tenapanor effectively reduced serum phosphate levels in multiple clinical trials and was generally well tolerated [78–80]. In a monotherapy trial, tenapanor administration lowered serum phosphorus in subjects from baseline concentrations of 8.1 mg/dL to 5.5 mg/dL in the efficacy analysis set at 12 weeks [80]. In a separate long-term study, tenapanor administration reduced serum phosphorus from baseline concentrations of 7.7 mg/dL to 5.1 mg/dL in the efficacy analysis set at 26 weeks [78]. A recent trial that compared the effectiveness of a combination of tenapanor and binder versus placebo and a binder showed in the primary analysis that tenapanor plus binder resulted in a 0.65 mg/dL (*p* < 0.001) larger mean serum phosphate reduction from the baseline compared to placebo plus binder [83]. Combination therapy with tenapanor and a binder resulted in significantly greater mean reductions from the baseline at all time points compared to treatment with a binder only (0.84–1.21 vs. 0.14–0.21 mg/dL, *p* < 0.001) [83]. In addition, almost twice as many patients treated with tenapanor and a binder achieved phosphate <5.5 mg/dL compared to patients treated with placebo and a binder (37–50% vs. 18–24%, *p* < 0.05) [83]. This dual-mechanism approach may be particularly relevant for patients with persistent hyperphosphatemia [83]. No effect on serum sodium, potassium, calcium, bicarbonate, chloride, glucose, or magnesium levels was observed in trials [81, 84].

Although not a part of the primary pathway of phosphate absorption in humans, NaPi2b may be another potential therapeutic target for novel hyperphosphatemia treatments. Clinical data for NaPi2b inhibitors are mixed. The investigational NaPi2b inhibitor ASP3325 reduced phosphate uptake in animal models [85], but no effect was observed in human trials [86]. The NaPi2b inhibitor DS-2330b was similarly unsuccessful for phosphate control in a human trial [87]. The NaPi2b inhibitor nicotinamide reduced mean phosphate levels in small trials of patients on dialysis [88, 89]. Recently, modified-release nicotinamide (250–1500 mg/d) was shown to be superior to placebo as an add-on therapy to phosphate binders in reducing serum phosphate concentrations in a large cohort of hemodialysis patients with hyperphosphatemia over the first 24 weeks of treatment (mean difference of −0.39 mg/dl), but the treatment effect was not maintained up to week 52. Several newly identified adverse events may have contributed to patients not tolerating long-term therapy.

In addition to drugs that address phosphate absorption, improvements can be expected in binder technology resulting in more potent and specific phosphate binding, smaller pill size, lower pill burden, and significantly lower side effects. For example, a phase 1 study evaluating lanthanum dioxycarbonate, a novel nanotechnology product that combines lanthanum with a potentially smaller pill size that is swallowed with water rather than chewed, was presented recently [90]. If available, newer binders could be combined with agents that address phosphate absorption to further improve the treatment of hyperphosphatemia and patient and provider experience.

## 5. Future Directions

Management of hyperphosphatemia remains a multidisciplinary effort that includes dietary counseling, an adequate dialysis dose, and pharmacological agents. New therapeutic innovations with novel mechanisms of action may increase the proportion of patients who achieve and maintain appropriate phosphate control. Clinicians should consider new hyperphosphatemia treatment paradigms that reflect the latest understandings of phosphate absorption to achieve phosphate concentrations <5.5 mg/dL (or closer to normal) consistently. If available for clinical use, one potential approach would be to use targeted paracellular phosphate absorption inhibitors alone or in combination with binders, if necessary, as part of a dual-mechanism pharmacological approach for patients with difficult to control hyperphosphatemia or for patients achieving phosphate goal concentrations but overburdened by pill numbers, side effects of high dose binders, or both. Improvements in binder technology may also result in more effective and better tolerated agents.

Thus, patients and providers experiencing “phosphate frustration” may get some relief when newer drug therapies become available. Such therapies would allow for improvements in the management of hyperphosphatemia, initially aiming at reaching and maintaining serum phosphate levels <5.5 mg/dL for most patients and potentially aiming at achieving normal phosphate levels in the future.

## Figures and Tables

**Table 1:** Efficacy of phosphate binders in phase 3 trials.

Reference	Number of patients	Duration of treatment (weeks)	Binders	Mean baseline phosphate levels (mg/dL)	Mean baseline phosphate levels at the end of treatment (mg/dL)	Percentage decrease in phosphate levels (%)
Emmett et al. [34]	91	2	Calcium acetate	7.3	5.9	19
			Placebo (comparator)	7.3	7.3	0

Floege et al. [35]	1041	24	Sucroferric oxyhydroxide	7.7	5.6	27
			Sevelamer (comparator)	7.4	5.3	28

Bleyer et al. [36]	84	8	Sevelamer hydrochloride	8.4	6.5	24
			Calcium acetate (comparator)	8.0	5.9	26

Mehrotra et al. [37]	126	8	Lanthanum carbonate	7.7	5.9	23
			Placebo (comparator)	7.4	7.9	(6)

Akebia therapeutics, data on file [38]	292	52	Ferric citrate	7.4	5.4	27
			Sevelamer and/or calcium acetate (comparator)	7.6	5.4	29

## Data Availability

No new data were generated or analyzed in support of this research.

## Additional Points

Despite low phosphorus diets, removal by dialysis, and phosphate binder use, many patients with CKD on dialysis are unable to consistently achieve and maintain serum phosphate concentrations <5.5 mg/dL, let alone the more aggressive target of normal phosphate levels (2.5–4.5 mg/dL). Phosphate binders can only remove approximately 300 mg of the estimated 2,500 mg daily dietary phosphate intake, and they do not directly target or act on either of the two main phosphate absorption pathways. Serum phosphate concentrations <4.5 mg/dL are associated with improved survival and cardiovascular outcomes, but real-world phosphate control data suggest that achieving and maintaining this goal would be extremely challenging using current therapies. The dominant paracellular phosphate absorption pathway is blocked by tenapanor, a novel sodium/hydrogen exchanger isoform 3 (NHE3) inhibitor that has been shown to effectively reduce phosphate levels in several trials and could be a very useful new drug to manage hyperphosphatemia in conjunction with existing therapies.
